# Microfluidic Assessment of Drug Effects on Physical Properties of Androgen Sensitive and Non-Sensitive Prostate Cancer Cells

**DOI:** 10.3390/mi12050532

**Published:** 2021-05-07

**Authors:** Da Luo, Na Liu, Yang Chen, Yan Peng, Tao Yue, Shan Cao, Yuanyuan Liu

**Affiliations:** 1 School of Mechatronics Engineering and Automation, Shanghai University, Shanghai 200444, China; ld18722273@shu.edu.cn (D.L.); arrowme@shu.edu.cn (Y.C.); pengyan@shu.edu.cn (Y.P.); tao_yue@shu.edu.cn (T.Y.); 2 Material Genome Institute, Shanghai University, Shanghai 200444, China; scao@shu.edu.cn

**Keywords:** physical properties, anti-cancer drug, high-throughput, microfluidic

## Abstract

The identification and treatment of androgen-independent prostate cancer are both challenging and significant. In this work, high-throughput deformability cytometry was employed to assess the effects of two anti-cancer drugs, docetaxel and enzalutamide, on androgen-sensitive prostate cancer cells (LNCaP) and androgen-independent prostate cancer cells (PC-3), respectively. The quantified results show that PC-3 and LNCaP present not only different intrinsic physical properties but also different physical responses to the same anti-cancer drug. PC-3 cells possess greater stiffness and a smaller size than LNCaP cells. As the docetaxel concentration increases, PC-3 cells present an increase in stiffness and size, but LNCaP cells only present an increase in stiffness. As the enzalutamide concentration increases, PC-3 cells present no physical changes but LNCaP cells present changes in both cell size and deformation. These results demonstrated that cellular physical properties quantified by the deformability cytometry are effective indicators for identifying the androgen-independent prostate cancer cells from androgen-sensitive prostate cancer cells and evaluating drug effects on these two types of prostate cancer.

## 1. Introduction

Prostate cancer is a commonly diagnosed cancer and one of the leading causes of cancer-related death in men [1]. The growth of prostate cancer is initially androgen-sensitive, and androgen deprivation therapy (ADT) has become a standard-of-care, first-line therapy for androgen-responsive prostate cancer. Unfortunately, most patients with prostate cancer eventually revert to all stages of androgen-independent growth, which presents a major clinical challenge in prostate cancer treatment [2,3,4]. The androgen-independent stage was commonly diagnosed through the monitoring of the prostate-specific antigen (PSA) concentration in patients who are under ADT, which can only identify the patient’s drug sensitivity status several months later. A timely identification of the patient’s sensitivity to androgen therapy is of great importance, in order for prostate cancer patients to adopt alternated drugs and ensure their survival rates.

Cellular physical properties are efficient label-free markers for determining cell states, metastatic potential and the degree of differentiation [5,6,7,8,9]. For example, cancer cells possess lower stiffness than normal cells from comparable regions [8,10], while cancer cells with different metastatic potential also possess different stiffness and size [11]. Some drugs can induce a change in the cell’s physical properties. For example, cytochalasin results in a decrease in the cell’s stiffness through depolymerizing actin filaments [12], while both docetaxel and cisplatin treatments cause an increase in the stiffness of the surviving prostate cancer cells [13]. These studies suggest that cellular physical properties are potential label-free markers for timely evaluating patients’ sensitivity to androgen therapy [14].

Recently, various techniques have been developed for quantifying the cellular physical properties, such as atomic force microscopy (AFM) [15], magnetic twisting cytometry [16], micropipette aspiration [17], nano/micropore devices [18,19] and optical stretching [20,21,22]. However, these approaches suffer from labor intensity and low-throughput measurement, which only characterizes a small number of cells in one experiment. In order to eliminate both the typical heterogeneities in biological specimens and the user’s own bias, the physical characterization for large-scale populations of cells is required [21,23]. The deformability cytometry first reported by Gossett, D. R. [24] is a high-throughput approach used for quantifying cellular stiffness and size in a single cell [25,26,27].

In this work, this microfluidic-based deformability cytometry method was employed to characterize the physical properties of androgen-insensitive prostate cancer cells PC-3 and androgen-sensitive cells LNCaP, which were treated using different concentrations of docetaxel and enzalutamide, respectively. Docetaxel is an anti-cancer drug that inhibits cancer cell proliferation and metastasis through promoting the tubules’ polymerization and their aggregation into stable microtubules. Enzalutamide is an androgen antagonist that inhibits prostate cancer cell proliferation through restraining the androgen’s bonding with the prostate cell. For each experimental condition, the stiffness and size of thousands cells were quantified. In this way, there was a sufficient amount of sample data to investigate the effects of these anti-cancer drugs on the proliferation of prostate cancer cell lines. The results showed that PC-3 cells were sensitive to docetaxel but not to enzalutamide, and LNCaP cells were sensitive to both drugs. In addition, changes in cell deformability and cell size are closely related to the drug concentration. The final stiffness of PC-3 and LNCaP both increased with the increase in docetaxel concentration, whereas only the PC-3 size increased while the size of most LNCaP had no significant change. In comparison, the stiffness and size of LNCaP changed with the increase in enzalutamide concentration, while the PC-3 showed no significant changes. These results demonstrated that physical properties can identify androgen-independent prostate cancer through treatment with the use of an androgen antagonist.

## 2. Materials and Methods

### 2.1. Device Setup and Working Principle

**Figure 1:**
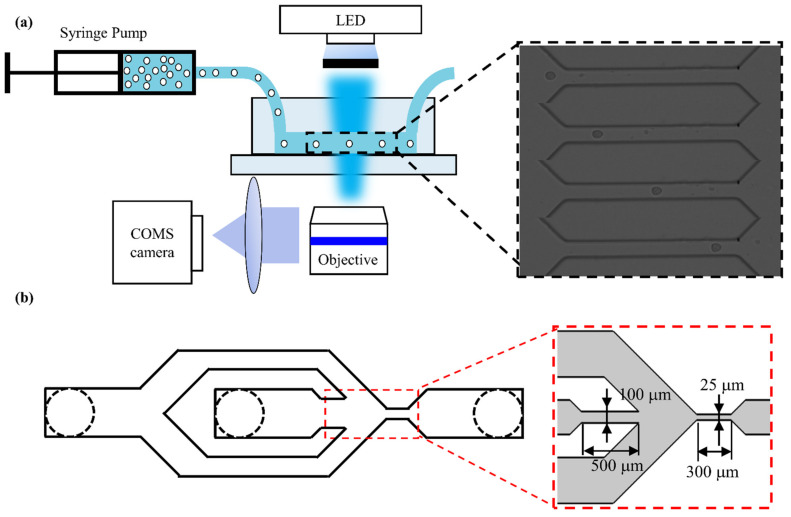
(**a**) The overview of the experimental system device and its detailed work schematic diagram and microfluidic channel geometry. The inset shows that cells flow through the constriction channel. (**b**) The improved microfluidic chip structure. The insets show the microchannel structure of the microfluidic chip.

As shown in Figure 1a, the utilized experimental setup integrated a self-developed microfluidic device, a high-speed CMOS camera (Phantom Miro R311, Vision Research, New Jersey, NJ, USA) and a microscope (Navitar, Rochester, NY, USA).

A simple microfluidic device with only one inlet and one outlet has been reported previously [28]. However, the cells passing through the microchannel could potentially collide with the wall, and, sometimes, dust and debris particles clog the narrow channel, which both have a negative effect on the experiment. To solve these problems, the device structures have been improved through adding sheath inlets on both sides of the chip sample inlet, as shown in Figure 1b. The sheath flows on both sides of the sample so that the cell suspension is in the middle of the microchannel and the cells are always in focus. To avoid any clogging of the microchannel, we have designed a micro column array with step-by-step filtering to be placed in the middle part of the channel, from the inlet to the microchannel. The gaps of the filtering array are 100 μm, 75 μm, 50 μm and 25 μm. The length of the microchannel where the cell is deformed is 300 μm. The size of the microchannel was designed to be 25 μm, which is slightly larger than the diameter of cancer cells (~16–20 μm), thus enabling the cells that were deformed by flow shear stress rather than the channel walls to pass through the channel. During measurement, the prepared cell suspension was pumped into the microfluidic chip using a syringe pump at a rate of 0.2 μL/s. The deformation process of cells was recorded using the high-speed camera at a frame rate of 5000 frames per second and an exposure time of 3 μs.

**Figure 2:**
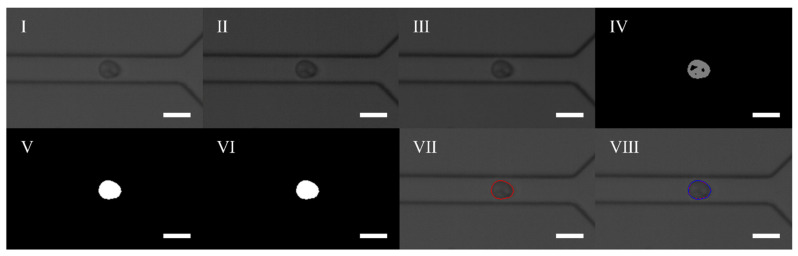
Image processing steps: (**I**). Defining ROI image; (**II**). Enhancing contrast ratio; (**III**). Filtering processing; (**IV**). The foreground image; (**V**). Filling the hole; (**VI**). Removing the noise points; (**VII**). The contour image; (**VIII**). The convex hull outline. The scale bar represents 25 μm.

A self-developed image processing algorithm was applied in order to measure both the deformation degree and physical properties of the cells. The processing steps are shown in Figure 2. Firstly, the ROI region was determined on the original image to improve the operation speed. Then the contrast of the ROI image was enhanced and smoothed through filtering. Afterwards, Gaussian mixture background modeling was used to extract and remove the background images in order to obtain the foreground. The rough foreground was then filled with holes to obtain the contour from the binary image. Finally, the contour image was processed using the convex hull to obtain the final cell contour. Here, we use the convex hull perimeter and the convex hull area to calculate the cell deformation according to Equation (1).

(1) $$D=1-\frac{4\pi A}{L^{2}}$$

where *A* is the area, *L* is the perimeter of the extracted cell and *D* is the deformation of the cell.

### 2.2. Fluid Simulation of Microchannels

**Figure 3:**
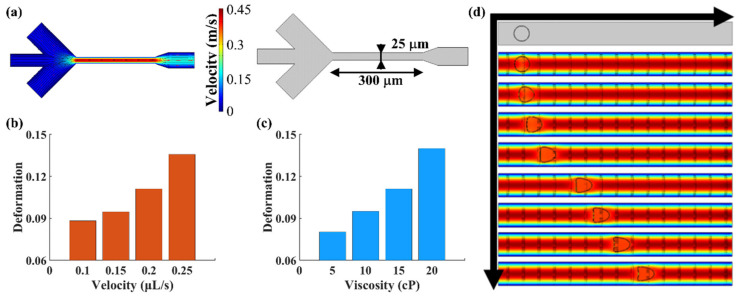
(**a**) The flow field distribution diagram and microchannel geometry. (**b**) Cell deformation under different fluid velocities with a fluid viscosity of 15 cP. (**c**) Cell deformation under different viscosities with a fluid velocity of 0.2 μL/s. (**d**) The process of cell deformation in the microchannel.

The deformation degree of cells in the microchannel is dependent on the fluid velocity and viscosity. Due to the optimization of microfluidic structure, we need to confirm the influence of new structure parameters before the experiment. Here, the COMSOL Multiphysics simulation software was used to analyze the effect of fluid velocity and viscosity on cell deformation. Figure 3a shows the flow field distribution diagram of the simulated microfluidic chip, where the black lines with arrows are the flow field lines. The middle channel on the left side of the chip is the cell suspension inlet and the two sides are the sheath inlets. In the experiment, the injected cells flowed through the constriction channel and were deformed by the flow shear stress with a peak velocity of 0.45 m/s. Figure 3b shows the cell deformation under different flow rates with a fluid viscosity of 15 cP. Figure 3c shows the cell deformation under different viscosities with a fluid velocity of 0.2 μL/s. Figure 3d shows the cell deformation at different flow times in the microchannel. As shown, the cells’ deformation degree increases with the increase in fluid viscosity, velocity and flow time in the microchannel.

### 2.3. Cell Culture and Sample Preparation

Androgen-independent prostate cancer cells (PC-3) and androgen-sensitive human prostate cancer cells (LNCaP) used in this report were purchased from ScienCell (Zhongqiaoxinzhou Biotech, Shanghai, China). PC-3 cells were cultured in F-12 K medium supplemented with 10% fetal bovine serum (Gibco, Waltham, MA, USA) and 1% penicillin/streptomycin (Gibco, Waltham, MA, USA) in a 5% CO2 humidified atmosphere at 37 °C. LNCaP cells were cultured in RPMI-1640 medium with 10% fetal bovine serum and 1% penicillin/streptomycin in a 5% CO_2_ humidified atmosphere at 37 °C.

Recent works evidenced the health status of cells is strongly related to the cell concentration and temperature [29]. To avoid the influence of these factors on the physical properties of cells, we cultured cells at the same conditions including cell concentration, temperature and time. The concentration of each type cell was the same as the control group after drug was added, which determined that changes in physical properties of cells was dependent on the effect of the drug.

To assess drug effects on physical properties of androgen sensitive and non-sensitive prostate cancer cells, PC-3 and LNCaP cells were treated with docetaxel (Selleck Chemicals, Houston, TX, USA) and enzalutamide (Selleck Chemicals, Houston, TX, USA). For docetaxel treatment, docetaxel was first dissolved in dimethyl sulfoxide (DMSO, Sigma-Aldrich, Saint Louis, MO, USA), and then diluted in a culture medium at the final concentrations of 1 μmol/L, 100 nmol/L, 10 nmol/L and 1 nmol/L in the respective samples. The culture medium was removed one day after the cell passage, and the new cell medium mixed with docetaxel was added. For enzalutamide treatment, cells were transferred to a medium supplemented with 10% carbon adsorption castrated fetal bovine serum (Gibco, Waltham, MA, USA)) a day before enzalutamide was added. All the samples were cultured in a 5% CO_2_ humidified atmosphere at 37 °C for 24 h.

For physical properties characterization, cells were resuspended using methylcellulose (MC, Sigma-Aldrich, Saint Louis, MO, USA) in phosphate buffer saline (PBS, HyClone, Chicago, IL, USA) at a concentration of 0.5% (*w*/*v*) to increase the shear viscosity.

### 2.4. Statistical Analysis

The extracted contour data of cells were analyzed using Origin2018 software. Measurement data were expressed as mean ± standard deviation (SD). When the *p* value was less than 0.05, it was considered statistically significant.

## 3. Results and Discussion

### 3.1. Physical Phenotyping of Prostate Cancer Cells PC-3 and LNCaP

**Figure 4:**
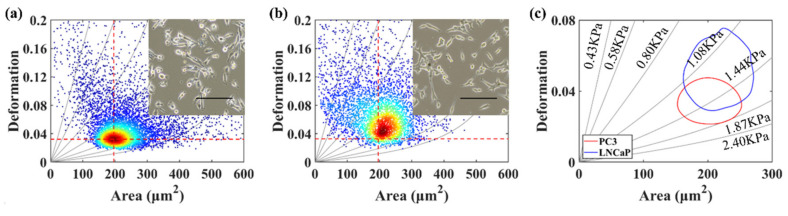
(**a**) The deformation versus area scatter plots of PC-3 cells. (**b**) The deformation versus area scatter plots of LNCaP cells. (**c**) The 50% density contour line of PC-3 and LNCaP cells. The red dotted lines for references represent the median of PC-3 cells’ cross-sectional area and deformation. The inset shows the optical microscopic images of PC-3 and LNCaP cells. The scale bar represents 200 μm.

In this work, the deformation and size of PC-3 and LNCaP cells were quantified under an injection velocity of 0.2 μL/s. The average deformation and area of androgen-independent prostate cancer cells PC-3 are 0.032 and 196 μm^2^ (Figure 4a), and these values are 0.045 and 212 μm^2^ for LNCaP cells (Figure 4b). Figure 4c shows the 50% density contour line of the PC-3 and LNCaP cells with iso-elasticity lines [28]. These lines showed the relationship amongst cell deformation, cell area and the cell’s elastic modulus [30]. It had been demonstrated that cell stiffness could be used as a marker for potential cell malignancy [31]. Considering cell size and deformation, PC-3 cells with higher invasiveness possess greater stiffness than LNCaP cells with lower invasiveness in our results.

### 3.2. Effect of Docetaxel on Cell Size and Deformation

**Figure 5:**
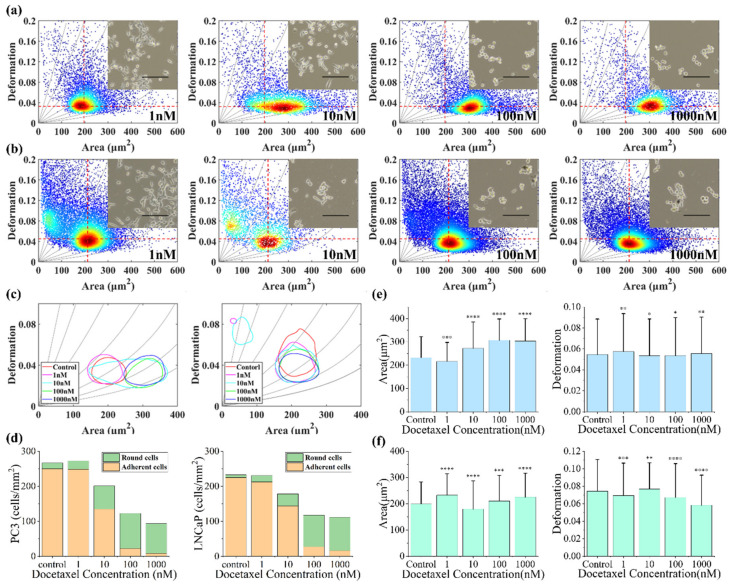
(**a**) The deformation versus area scatter plots of PC-3 cells cultured in cell medium containing different docetaxel concentrations after 24 h. (**b**) The deformation versus area scatter plots of LNCaP cells cultured in cell medium containing different docetaxel concentrations after 24 h. The order is 1 nmol/L, 10 nmol/L, 100 nmol/L and 1 μmol/L. (**c**) The 50% density contour line of different docetaxel concentrations on PC-3 and LNCaP cells. (**d**) Quantity trends of round cells and adherent cells in PC-3 and LNCaP cells. (**e**) The average degree of PC-3 cell area and deformation under docetaxel. (**f**) The average degree of LNCaP cell area and deformation under different docetaxel concentrations. *, **, *** and **** indicate *p* values of less than 0.05, 0.01, 0.001 and 0.0001, respectively. The red dotted lines for references represent the median of the control group PC-3 and LNCaP cells’ cross-sectional area and deformation. The inset shows the optical microscopic images of untreated control group and docetaxel-treated cells. The scale bar represents 200 μm.

Studies have demonstrated that docetaxel can induce cell morphologic changes, reduce cell adhesion and inhibit cell proliferation [32]. In order to further understand the effect of docetaxel on the physical properties of prostate cancer cell lines, we measured the size and deformation of PC-3 and LNCaP cells cultured in a medium with different docetaxel concentrations (1 μmol/L, 100 nmol/L, 10 nmol/L and 1 nmol/L respectively) for 24 h, and compared them with an untreated control group. Figure 5 shows the effects of docetaxel on PC-3 cells and LNCaP cells.

The results showed that deformation and size of cells were both closely related to docetaxel concentration. In addition, PC-3 cells change mainly in area while LNCaP cells change in deformation under the function of docetaxel (Figure 5a,b). As shown in Figure 5c, the cell area and deformation of PC-3 were slightly reduced under low drug concentrations (1 nmol/L). This might be due to the fact that the cells were in a stage of microtubule aggregation under the function of the drugs, and the microtubule network inside the cells had not been stabilized. When the concentration of the drug was increased, the cell size and cell stiffness were increased because of the stabilization of their internal structure. Most of PC-3 cells began to increase in area when the docetaxel concentration was 10 nmol/L, indicating that the concentration of the drug significantly affecting PC-3 cells. For LNCaP cells, the cell deformation gradually decreased with the increase in drug concentration (Figure 5c). This may be because under lower concentrations (1 nmol/L, 10 nmol/L), the cells were still in the stage of adaption to the drug’s function and in order to be stable at a higher concentration. Interestingly, when the concentrations of docetaxel were 10 nmol/L and 1 nmol/L, the scatter plots of LNCaP cell deformation–area showed two focal regions after 24 h (Figure 5b). At this point, the left region with a small area and large deformation should be the apoptotic cells and fragments under the function of the drug, whereas the right region with a large area and small deformation should be the parts of the cells that survived and adapted to the drug. The changes in cell area and deformation were most obvious at 10 nmol/L, which showed that the drugs had an obvious effect on the cells, and would help us to quickly find the sensitive concentration of the drug. In addition, more and more cells exfoliated and the cell number decreased with the increase in docetaxel concentrations (Figure 5d), which showed that the cellular states were deteriorating. As shown in Figure 5e,f, the average area and deformation of PC-3 and LNCaP cells under different docetaxel concentrations were compared. Evidently, the size and deformation of PC-3 and LNCaP cells were significantly different compared to the control group. After taking into account the changes in cells’ area and deformation, in combination with the iso-viscoelastic lines, PC-3 and LNCaP cells hardened with an increased docetaxel concentration. This demonstrated the value of the cytoskeleton in changing the physical properties of cancer cells, as docetaxel inhibited the tubules’ polymerization and aggregation into stable microtubules, resulting in an increase in cell stiffness. This is consistent with other previous studies [7,33].

### 3.3. Effect of Enzalutamide on PC-3 Cell Size and Deformation

**Figure 6:**
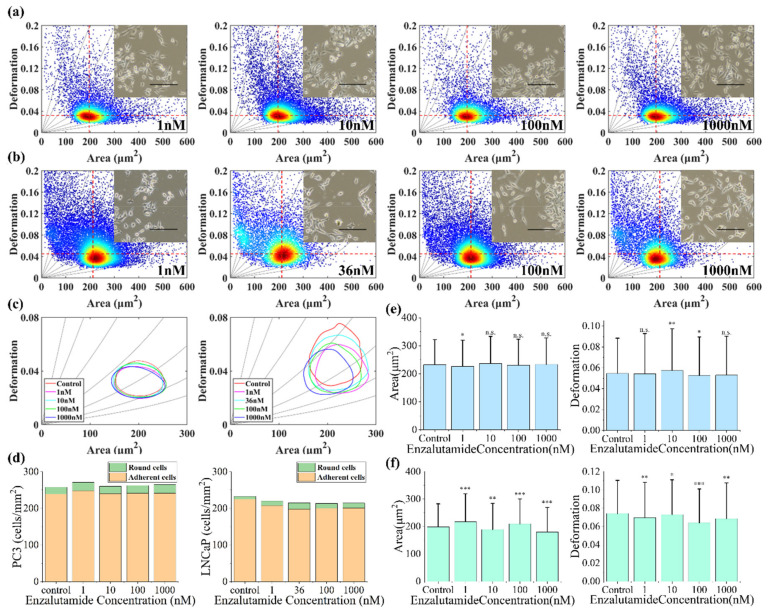
(**a**) The deformation versus area scatter plots of PC-3 cells cultured in cell medium containing different enzalutamide concentrations after 24 h. (**b**) The deformation versus area scatter plots of LNCaP cells cultured in cell medium containing different enzalutamide concentrations after 24 h. The order is 1 nmol/L, 10 nmol/L, 100 nmol/L and 1μmol/L. (**c**) The 50% density contour line of different enzalutamide concentrations on PC-3 and LNCaP cells. (**d**) Quantity trends of round cells and adherent cells in PC-3 and LNCaP cells. (**e**) The average degree of PC-3 cell area and deformation under different enzalutamide concentrations. (**f**) The average degree of LNCaP cell area and deformation under different enzalutamide concentrations. The *, **, *** and n.s. indicate *p* values of less than 0.05, 0.01, 0.001, 0.0001 and more than 0.05, respectively. The red dotted lines for references represent the median of the control group PC-3 and LNCaP cells’ cross-sectional area and deformation, respectively. The inset shows the optical microscopic images of untreated control group and enzalutamide-treated prostate cancer cell line. The scale bar represents 200 μm.

Studies have shown that the growth rate of androgen-independent cells in the medium lacking hormones is significantly faster than that of androgen-dependent prostate cancer cells [32]. PC-3 is an androgen-independent cell line while LNCAP is an androgen-dependent cell line. In order to understand the effects of enzalutamide on the physical properties of prostate cancer cell lines PC-3 and LNCaP, we further measured the changes in deformation and size of PC-3 and LNCaP cells after they were cultured in both the control group and the different concentrations of the enzalutamide (1 μmol/L, 100 nmol/L, 10 nmol/L and 1 nmol/L respectively) medium for 24 h. Figure 6 shows the effects of enzalutamide on PC-3 cells and LNCaP cells.

The results showed that PC-3 cells treated with different concentrations of enzalutamide for 24 h did not have significant changes in cells’ deformation and area compared to the control group (Figure 6a,c). This is because enzalutamide is an androgen antagonist, which can inhibit the binding of androgen to its receptor, whereas PC-3 is an androgen-insensitive prostate cancer cell. Therefore, from the perspective of the drug mechanism, enzalutamide does not affect the morphology and growth of PC-3 cells, proven by the results of the experiment. Conversely, the deformation and area of LNCaP cells were closely related to the concentration of enzalutamide, and the cell area and deformation gradually decreased with an increase in drug concentration (Figure 6b,c). The number of round cells and adherent cells did not change significantly with the increase in enzalutamide concentrations (Figure 6d). As shown in Figure 6e,f, the average area and deformation of PC-3 and LNCaP cells under different enzalutamide concentrations was compared. Clearly, the area and deformation of PC-3 were both slightly different compared to the control group, while LNCaP cells were significantly different compared to the control group. Considering the changes in cells’ area and deformation, combined with iso-viscoelastic lines, the stiffness of LNCaP cells increased at a concentration of 1 nmol/L and 36 nmol/L and tended to decrease at a concentration of 100 nmol/L and 1 μmol/L as the cells adapted to the influence of the drug, which indicated the process of LNCaP cells adapting to the environment and gradually converting into androgen-independent cells under the condition of androgen removal. Overall, PC-3 cells had no changes in stiffness while LNCaP cells hardened with the treatment of enzalutamide.

## 4. Conclusions

In this work, we have employed high-throughput deformability cytometry to assess the physical effects of two anti-cancer drugs, namely docetaxel and enzalutamide, on both androgen-independent human prostate cancer cells PC-3 and androgen-sensitive prostate cancer cells LNCaP with a throughput of more than 1000 cells/s. The quantified results show that PC-3 and LNCaP present physical responses to the same anti-cancer drug. With an increase in docetaxel concentration, PC-3 cells present an increase in stiffness and size while LNCaP cells only present an increase in stiffness. However, PC-3 cells present no physical changes, whereas LNCaP cells present changes in both cell size and deformation with an increase in enzalutamide concentration. In addition, changes in cell deformability and cell size are closely related to the drug concentration and duration of treatment. The changes in the cells are different with different drug treatments, and tend to stabilize at a certain concentration. These results demonstrated that cellular physical properties quantified by the deformability cytometry are effective indicators for identifying the androgen-independent prostate cancer cells from androgen-sensitive prostate cancer cells and evaluating drug effects on these two types of prostate cancer.

## Data Availability

The data presented in this study are available in Supplementary Material.

## Supplementary Materials

The following are available online at https://www.mdpi.com/article/10.3390/mi12050532/s1.
